# Bacteriophage-Host Association in the Phytoplasma Insect Vector *Euscelidius variegatus*

**DOI:** 10.3390/pathogens10050612

**Published:** 2021-05-17

**Authors:** Marta Vallino, Marika Rossi, Sara Ottati, Gabriele Martino, Luciana Galetto, Cristina Marzachì, Simona Abbà

**Affiliations:** 1 Institute for Sustainable Plant Protection, National Research Council of Italy, Strada delle Cacce 73, 10135 Torino, Italy; marika.rossi@ipsp.cnr.it (M.R.); sara.ottati@ipsp.cnr.it (S.O.); gabriele.martino@ipsp.cnr.it (G.M.); luciana.galetto@ipsp.cnr.it (L.G.); cristina.marzachi@ipsp.cnr.it (C.M.); simona.abba@ipsp.cnr.it (S.A.); 2 Dipartimento di Scienze Agrarie, Forestali ed Alimentari DISAFA, Università degli Studi di Torino, Largo Paolo Braccini 2, 10095 Grugliasco, Italy

**Keywords:** microbiome, bacteriophages, insect vectors, *Euscelidius variegatus*, phytoplasma, Flavescence doreé, transcriptome, electron microscopy

## Abstract

Insect vectors transmit viruses and bacteria that can cause severe diseases in plants and economic losses due to a decrease in crop production. Insect vectors, like all other organisms, are colonized by a community of various microorganisms, which can influence their physiology, ecology, evolution, and also their competence as vectors. The important ecological meaning of bacteriophages in various ecosystems and their role in microbial communities has emerged in the past decade. However, only a few phages have been described so far in insect microbiomes. The leafhopper *Euscelidius variegatus* is a laboratory vector of the phytoplasma causing Flavescence dorée, a severe grapevine disease that threatens viticulture in Europe. Here, the presence of a temperate bacteriophage in *E. variegatus* (named Euscelidius variegatus phage 1, EVP-1) was revealed through both insect transcriptome analyses and electron microscopic observations. The bacterial host was isolated in axenic culture and identified as the bacterial endosymbiont of *E. variegatus* (BEV), recently assigned to the genus *Candidatus* Symbiopectobacterium. BEV harbors multiple prophages that become active in culture, suggesting that different environments can trigger different mechanisms, finely regulating the interactions among phages. Understanding the complex relationships within insect vector microbiomes may help in revealing possible microbe influences on pathogen transmission, and it is a crucial step toward innovative sustainable strategies for disease management in agriculture.

## 1. Introduction

Like all other organisms, insects harbor a rich, dynamic, and interactive community of microorganisms, collectively known as the microbiome, which comprises not only living members (microbiota), but also elements considered as not living organisms (viruses, plasmids, prions, viroids, and free DNA) and a whole spectrum of molecules produced by the microorganisms [1]. Both microbiome composition and its modification influence insect ecology, physiology, evolution, and behavior through genetic and metabolic interactions. In many cases, an insect’s survival depends on its microbiome composition [2]. For these reasons, both pest control and insect protection may take advantage by deciphering the relationships between insects and their microbiome as well as among microbiome components [3].

In the past decade, many studies have been devoted to characterizing the microbiome of insects, essentially through next-generation sequencing approaches. Even though bacteria, fungi, protozoa, and viruses may be associated with their insect host permanently or transiently, the vast majority of these works have focused on bacterial communities [2,4]. However, more recently, the study of insect virome (inclusive of viruses that infect eukaryotic cells, bacteriophages that infect bacteria, viruses that infect archaea, and virus-derived genetic elements within host chromosomes) is gaining more attention [5,6,7,8,9,10,11].

Bacteriophages are the most abundant organisms in the biosphere [12]. Interest in studying phages has recently increased in medicine because of their ability to shape the composition and diversity of the human gut microbiome [13,14], in pathogen control and their possible use as an alternative to antibiotics (phage therapy) [15,16], and in ecology because of the important role they play in different ecosystems [17,18]. Nevertheless, only a minimal part of phage biodiversity has been described [18,19].

In the case of insect microbiomes, few phages have been identified, and their impact on insect biology is still poorly understood. The arthropod endosymbionts *Spiroplasma* spp., *Candidatus* Hamiltonella defensa, and *Wolbachia* spp. are known to host bacteriophages [20,21,22]. The best characterized insect-bacteria-phage association is the tripartite interaction among the pea aphid *Acyrthosiphon pisum*, the insect endosymbiont *Hamiltonella defensa*, and its bacteriophage named APSE-1 [21]. Aphids carrying APSE-1 are more resistant against parasitoid attacks compared to aphids without this phage, thanks to eukaryote-targeted toxins encoded by the phage genome [23,24]. The symbiosis system comprising eukaryotic hosts, bacterium Wolbachia, and bacteriophages WO is widely spread through nearly half of the known arthropod species [25]. WO has received heightened interest because of its ability to mediate horizontal transfer of Wolbachia bacterial genes [26] and its possible involvement in the cytoplasmic incompatibility in insect hosts induced by Wolbachia [27,28]. Since their discovery and despite their ecological relevance, these cases remain the only characterized bacteriophage-endosymbiont interactions described in insects.

Here, we report the identification of bacteriophages in the phytoplasma insect vector *Euscelidius variegatus* Kirschbaum. Phytoplasmas are plant pathogenic bacteria transmitted by insects that can cause severe loss in agriculture. The leafhopper *E. variegatus* (Cicadellidae: Deltocephalinae) is a multivoltine and polyphagous species, widespread in Europe and North America. *E. variegatus* is a natural vector of ‘*Candidatus* Phytoplasma asteris’ (chrysanthemum yellows strain) and a laboratory vector of the Flavescence dorée phytoplasma (reviewed in [29]), and it is used as a model system to study phytoplasma-vector interactions because of the difficulties in rearing the monovoltine natural vector *Scaphoideus titanus*. Insecticide treatments are the main control strategy to contain disease spread by insect vectors [30]. Recent findings proved that the microbiome can interfere with the vector ability to acquire and transmit pathogens directly or indirectly [4,31,32,33]. Gong et al. [34] showed that the artificial association of the insect vector *Nilaparvata lugens* with a Wolbachia bacterial strain from another leafhopper species makes the insect unable to transmit a severe viral disease of rice. This work demonstrates that manipulating insect microbiome is a viable strategy for changing insect traits and that a better knowledge of insect microbiome is the basis for new plant pest control strategies.

In this work, the presence of a temperate bacteriophage in *E. variegatus* (named Euscelidius variegatus phage 1, EVP-1) was revealed through both insect transcriptome analyses and microscopic observations. The bacterial host was isolated in pure culture and identified as the bacterial endosymbiont of *E. variegatus* (BEV), which has been recently assigned to the genus *Candidatus* Symbiopectobacterium [35]. Actually, BEV harbors multiple prophages that become active in culture, suggesting that different environments can trigger different mechanisms that finely regulate the within-host interactions among phages. Moreover, our results demonstrate that identifying expressed phage sequences in transcriptomic data can be a new and valuable approach to detect and study bacteriophages.

## 2. Results

### 2.1. Bacteriophage-Like Particles in Euscelidius variegatus

**Figure 1:**
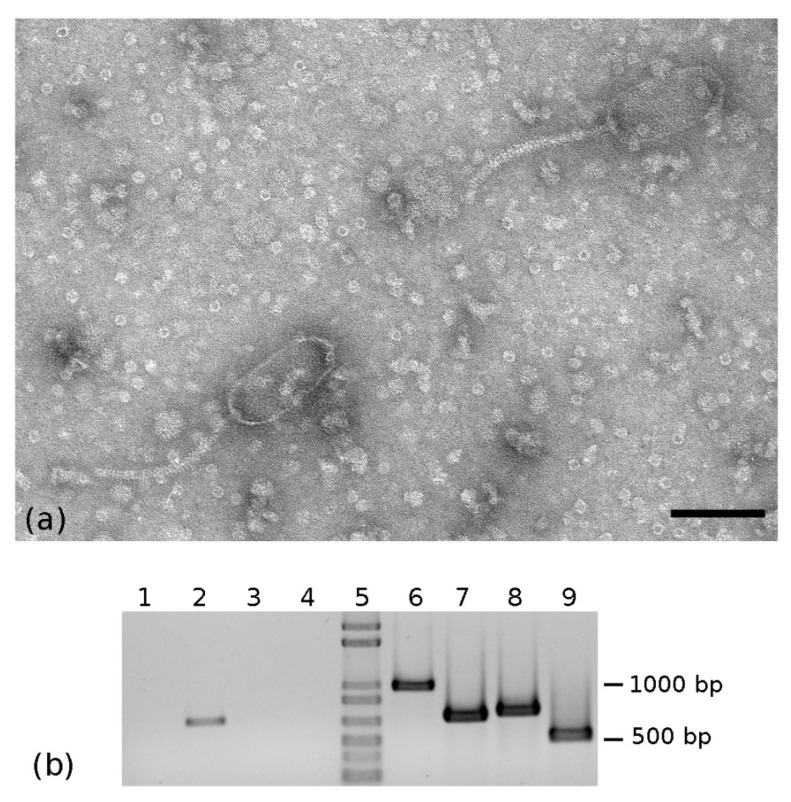
(**a**) TEM micrograph of siphovirus-like particles observed in negatively stained partial viral purification from *Euscelidius variegatus* Torino population; bar = 100 nm. (**b**) PCR on the DNA extracted from viral particles (Lanes 1–4) and *E. variegatus* whole insect (Lanes 6–9). Lanes 1,6: PCR with BEV3/4 primers; Lanes 2,7: PCR with EvaTO_phage1 primers; Lanes 3,8: PCR with EvaTO_phage2 primers; Lanes 4,9: PCR with EvaTO_phage3 primers. Lane 5: 1 Kb Plus DNA Ladder (Invitrogen).

During the electron microscope observation of a partial viral purification obtained from a *Euscelidius variegatus* Torino (EvaTO) population [36]*,* aimed at revealing insect virus particles, bacteriophage-like particles were serendipitously observed. Those phages had head-and-tail morphology typical of the viral order *Caudovirales* and in particular of the *Siphoviridae* family; they had a prolate (elongated) oval head 132 nm long (SE = 0.7 nm; n = 48) and 59 nm wide (SE = 0.3 nm; n = 48) and a flexuous thin non contractile tail 179 nm long (SE = 4.4 nm; n = 16) (Figure 1a). Frequently, heads and tails appeared detached (not shown).

### 2.2. Selection of Expressed Bacteriophage Sequences

In an effort to identify the siphovirus-like phage observed by TEM, RNA extracted from EvaTO adults was used to construct two cDNA libraries. The two datasets were then merged and depleted of reads matching to the insect transcriptome in order to generate a *de novo* metatranscriptome assembly of *E. variegatus* microbiome. A blastx analysis of the assembled sequences revealed the presence of 12 expressed sequences that were identified as hallmark bacteriophage structural genes, i.e., major capsid, minor capsid, baseplate, major tail, minor tail, portal, tail fiber, tail sheath, collar, and head-tail joining proteins. After a further annotation with the ViPTree server [37], 5 out of 12 sequences appeared to be polycistronic mRNAs (Table 1), i.e., they encoded at least two putative proteins.

The selected transcripts included three different complete major capsid/head proteins, suggesting that the selected expressed sequences belonged to at least three different phages, thereafter referred to as EVP-1 (Euscelidius variegatus phage 1, associated with MW965291 ORF3), EVP-2 (associated with MW965292) and EVP-3 (associated with MW965289 ORF2).

### 2.3. Phylogenetic Analysis of the Identified Major Capsid Proteins

**Figure 2:**
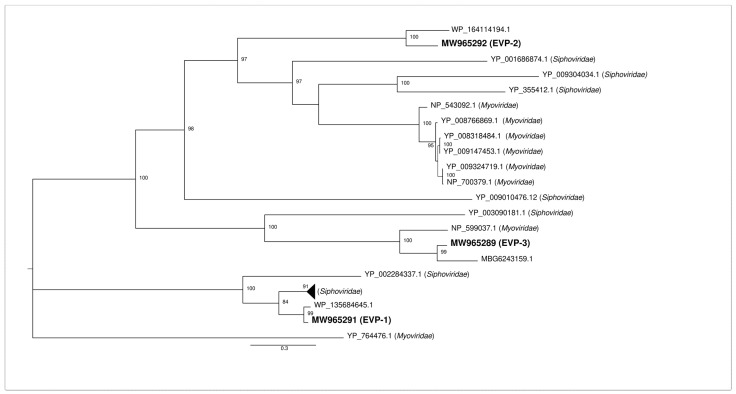
Phylogenetic analysis of the deduced amino acid sequences of the three identified major capsid proteins. Major capsid proteins were aligned by MUSCLE and the phylogeny was inferred by the maximum-likelihood method using the CIPRES Science Gateway V. 3.3. Bootstrap analyses with 1000 iterations were used with the Dayhoff substitution matrix to estimate pairwise distances. Only bootstrap values higher than 70 are shown. The three identified major capsid proteins are in bold. As regards MW965289 and MW965291, which are both polycistronic transcripts, only the deduced amino acid sequences corresponding to the major capsid proteins were included in the alignment, i.e., ORF2 and ORF3, respectively. The scale bar indicates the evolutionary distance for 0.3 amino acid substitutions per site. The collapsed branch grouped nine sequences: BCI49740.1, Stx2a-converting phage Stx2 EH2201; BCI49860.1, Stx1a-converting phage Stx1 EH199; QIW91675.1, Escherichia phage Lys8385Vzw; YP_001449293.1, Enterobacteria phage BP-4795; YP_009909243.1 Enterobacteria phage 2851; YP_002274188.1, Enterobacteria phage YYZ-2008; BAT31949.1, Stx2-converting phage Stx2a F349; YP_009909397.1, Stx2-converting phage Stx2a WGPS6; and YP_009909320.1, Stx2-converting phage Stx2a WGPS8. GenBank accession numbers of the other major capsid proteins are: WP_135684645.1, *Klebsiella pneumoniae*; YP_002284337.1, Pseudomonas phage PAJU2; WP_164114194.1, *Serratia marcescens*; YP_009324719.1, Salmonella phage 118970 sal3; NP_700379.1, Salmonella phage ST64B; YP_008766869.1, Shigella phage SfIV; YP_009147453.1, Enterobacteria phage SfI; YP_008318484.1, Shigella phage SfII; NP_543092.1, Enterobacteria phage phiP27; YP_355412.1, Burkholderia phage Bcep176; YP_001686874.1, Azospirillum phage Cd; YP_009304034.1, Brucella phage BiPBO1; YP_009010476.1, Geobacillus phage GBK2; NP_599037.1, Enterobacteria phage SfV; YP_003090181.1, Burkholderia phage KS9; YP_764476.1, Geobacillus phage GBSV1; MBG6243159.1, *Candidatus* Symbiopectobacterium sp. Dall1.0.

The deduced amino acid sequences of the three major capsid proteins were aligned to the first hit retrieved by blastx against the NCBI nr database and the first ten hits identified by blastp against the NCBI RefSeq protein limited to the taxon “Viruses (taxid:10239)”. We considered for the analysis only complete major capsid proteins from phages assigned to one of the nine *Caudovirales* families recognized by the International Committee on Taxonomy of Viruses (ICTV). The phylogenetic analysis (Figure 2) showed that EVP-1 was part of a completely separate cluster from the one that included the other two identified major capsid proteins. Such a cluster was formed only by major capsid proteins from members of the family *Siphoviridae*. The other cluster was far more heterogeneous, including members of both the families *Siphoviridae* and *Myoviridae.* EVP-2 and a protein identified in *Serratia marcescens* (best blastx hit) formed a separate branch that diverged from a clade including both siphoviruses and myoviruses. Finally, EVP-3 formed a strongly supported clade (100% bootstrap value) with major capsid proteins from *Candidatus* Symbiopectobacterium sp. Dall1.0 and from a member of the family *Myoviridae.*

### 2.4. Detection and Prevalence of the Three Phages

Specific primers designed on the three genes (Table 2), coding for the identified major capsid proteins, were used on DNA extracted from insect whole bodies and a partial viral purification. All the three primer pairs produced amplicons of the expected size and sequence from 20 individuals randomly selected from the EvaTO population (data not shown). By contrast, only primers designed on EVP-1 major capsid protein gave an amplification product from the DNA extracted from viral particles (Figure 1b). Therefore, we could reasonably associate the phage observed by TEM to EVP-1.

### 2.5. Isolation of EVP-1 Bacterial Host

In an attempt to identify the EVP-1 bacterial host, EvaTO hemolymph was cultivated on chocolate agar and purple agar plates, yielding several colonies (Figure S1). Few colonies appeared after one/three days of incubation and showed a fast growth. After 7-10 days, many other whitish small colonies (almost forming a layer) appeared; they looked all similar and grew slowly (Figure S1a,b). The fast growing (1C-3C, 5C-7C, 1P, 2P) and some of the slow growing (4C, 8-15C, 3-5P) colonies were isolated and sub-cultivated on both chocolate and purple agar, irrespective of their original selection medium. Ten out of the 20 isolated colonies were positive to the PCR amplification with EVP-1 primers (Figure S1b). All the fast-growing colonies were negative; almost all the slow growing colonies (except 10C and 11C) were positive and they were considered as EVP-1 bacterial hosts. The growth of EVP-1 positive colonies on chocolate and purple agar was scanty, with low viability. Attempts at subculturing these colonies on Tryptic soy agar (TSA) resulted in faster growth, with similar viability (about two weeks). Three colonies were selected and maintained on TSA for further investigations: 4C, 12C, and 14C. At each subculture step, the presence of EVP-1 was checked by PCR with EvaTOphage1 primers (not shown). The 16S ribosomal sequences of the three colonies were 100% identical to each other and 99% identical (95% query coverage) to the one isolated from the “Bacterial parasite of *Euscelidius variegatus*” (BEV, GenBank accession number: Z14096 [38]) (Figure S2), which has been recently renamed Candidatus *Symbiopectobacterium* [35]. Based on this result, primers BEV3/BEV4 [39], specifically designed on the BEV 16S ribosomal sequence, were used hereinafter. In particular, they were used to exclude the presence of bacterial host DNA after DNA extractions from viral particles (Figure 1b).

**Figure 3:**
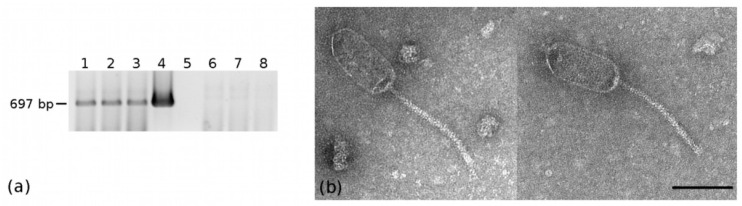
(**a**) PCR and RT-PCR experiments on *E. variegatus* dissected guts with EvaTO_phage1 primers. Lanes 1–3: RT-PCR on RNA extracted from three pools (five samples each) of *E. variegatus* guts. Lanes 6–8: PCR on the same RNA sample as in lanes 1–3 without the retrotranscription step. Lane 4: PCR on DNA extracted from a pool of five *E. variegatus* guts. Lane 5: negative control (no DNA) (**b**) TEM micrograph of siphovirus-like particles observed in negatively stained crude extract from dissected gut of *E. variegatus* Torino; bar = 100 nm.

Previous literature has reported that BEV can colonize multiple insect host organs, including the midgut [40,41]. PCR and RT-PCR experiments on *E. variegatus* dissected guts not only confirmed the presence of EVP-1 but also revealed that the phage was transcriptionally active in this tissue (Figure 3a). In addition, TEM observations of negatively stained crude extract from guts demonstrated the presence of siphovirus-like phage particles similar to those observed in the viral purification from the whole insect (Figure 3b).

### 2.6. Multiple Phages in EVP-1 Host

**Figure 4:**
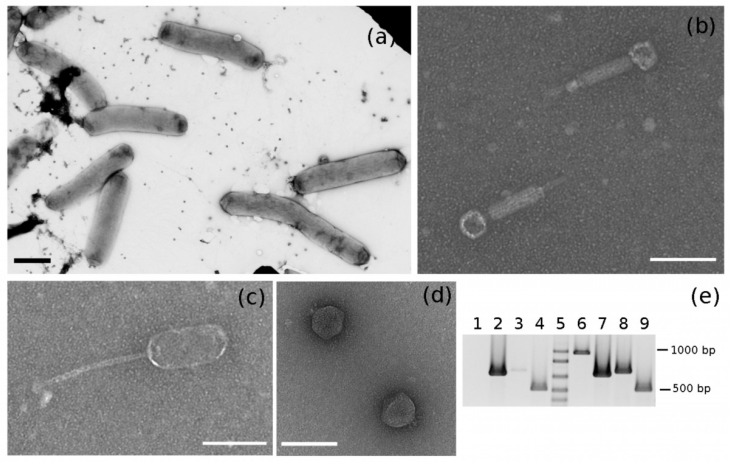
TEM micrographs of the negatively stained 4C colony showing bacterial cells (**a**), myovirus-like particles (**b**), siphovirus-like particle (**c**), and podovirus-like particles (**d**). Scale bars correspond to 1 μm (**a**) or 100 nm (**b**–**d**). (**e**) PCR on DNA extracted after the enrichment of viral particles from bacterial colony 4C (Lanes 1,4) and PCR on the bacterial colony 4C (Lanes 6–9). Lanes 1,6: PCR with BEV3/BEV4 primers; Lanes 2,7: PCR with EvaTO_phage1 primers; Lanes 3,8: PCR with EvaTO_phage2 primers; Lanes 4,9: PCR with EvaTO_phage3 primers. Lane 5: 1 Kb Plus DNA Ladder (Invitrogen).

According to a preliminary survey of BEV genome size and content [42], there was evidence for an extrachromosomal element that could represent a prophage and 65 partial coding sequences that could be ascribed to phages. A blastn analysis against all the BEV sequences submitted to the GenBank Trace Archive revealed that 11 out of the 12 putative phage transcripts identified in this work found a hit with percentages of identity >88%, but with low percentages of query coverage (average coverage < 41%) (Table S1). Such result could be explained by the fact that some deposited sequences are shorter than the identified transcripts and all of them contain ambiguous nucleotides (Ns), which in the case of gnl|ti|2292005461 represented more than 50% of its length. Nevertheless, those results suggested that most of the identified sequences could be assigned to phages that have BEV as their bacterial host. A colony PCR confirmed that EVP-2 and EVP-3 were also likely to be associated with BEV (Figure 4e).

**Figure 5:**
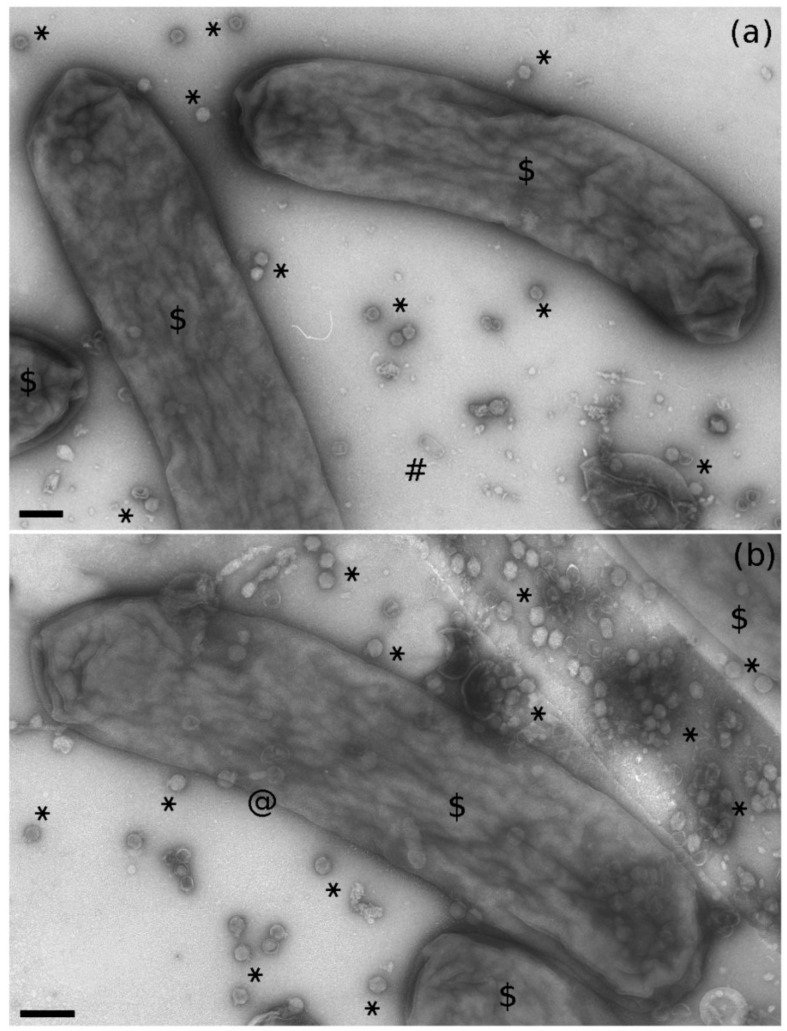
TEM micrographs of a negatively stained portion of the 4C colony showing bacterial cells (**a**,**b**, $), podovirus-like particles (**a**,**b**, asterisks), a siphovirus-like particle without tail (**a**, #), a myovirus-like particle with contracted tail (**b**, @). Bars represent 200 nm.

Electron microscopy observation of negative-stained bacteria from TSA cultures revealed rods from 2.1 to 5.3 μm long (with a mean value of 3.1 μm, n = 60), from 0.60 to 0.79 μm wide (with a mean value of 0.68 μm, n = 60), without flagella and sometimes curved (Figure 4a). In the proximity of bacterial cells, some bacteriophage particles were noted; some were siphovirus-like and similar to those observed in the insect extract (Figure 4c). Moreover and unexpectedly, myovirus-like (Figure 4b) and podovirus-like (Figure 4d) particles were also present. In particular, the number of podovirus-like particles were approximately 100-times higher than the number of siphovirus- and myovirus-like ones (Figure 5).

The bacterial colony was subjected to an enrichment of viral particles and a DNase treatment prior to DNA extraction to ensure the purification of encapsidated DNA only. Given the different phage morphologies observed by TEM, the three phage primer pairs were used in PCR. Amplicons of the expected size were obtained with specific primers for EVP-1 and EVP-3, whereas a faint band was observed with EVP-2 primers (Figure 4e). BEV primers failed to amplify bacterial DNA, so we could exclude the presence of contaminating phage DNA integrated into the host chromosome. PCR results confirmed that the siphovirus-like phage observed by TEM was EVP-1 and suggested that the myovirus-like particles could be associated with EVP-3.

## 3. Discussion

Phage “omics” studies are represented mostly by metagenomic shotgun analyses applied to a wide range of environments and locations, from uncultured marine samples [43,44,45] to human gut microbiome [44,46,47,48,49]. Differential filtrations and density-dependent gradient centrifugations are the usual enrichment steps taken to concentrate viral DNA and limit bacterial constituents and other contaminants before sequencing.

While metagenomics is the most rapid and efficient approach used to describe the overwhelming diversity of phages, transcriptomics has been generally used to investigate the transcriptional response of bacteria isolated in pure cultures upon phage infection [50,51,52]. Only a few untargeted metatranscriptomic studies, which explored mainly soil microbial communities, reported the discovery of novel RNA phages [53,54] and phage-related mRNA sequences [55,56].

Metatranscriptomics has here been integrated with classical microbiological and microscopy techniques to identify a DNA tailed-phage and its bacterial host within the microbiome of a phytoplasma insect vector. To this end, we re-analyzed two RNA-seq libraries that were originally constructed without any prior phage enrichment step to explore *E. variegatus* transcriptome [57]. *De novo* identification of phage sequences can be an extremely challenging task, especially from a background of genes expressed by the insect host and all the active microorganisms that constitute its microbiome. Nevertheless, the stringent selection of phage-hallmark genes resulted in a reliable identification of the observed phage. Such a bioinformatic approach was clearly a non-exhaustive way to retrieve all the expressed phage sequences in the libraries. Some were probably overlooked due to the lack of similarity with known phage sequences (the so-called “dark matter”) and/or the wrongful identification as bacterial genes in public databases. The absence of a biomarker gene among DNA phages and the polyphyletic origin of most viral lineages pose a hindrance for identifying all the putative phages. In any case, the characterization of the whole *E. variegatus* phageome was beyond the scope of this work, and the chosen approach proved effective in the detection of the transcribed phage genes associated with the observed phage particles.

Although RNA-seq data provided information about the active fraction of phages within the insect microbiome, they were not sufficient to apply the new computational approaches developed to predict phage-host relationships [58,59]. Therefore, classical microbiological techniques were applied for the unambiguous phage-host identification. All the bacterial hosts of the phages identified by the BLAST analyses belonged to the order *Endobacterales*, so it was reasonable to hypothesize that bacteria of the same order could be part of the *E. variegatus* microbiome and hosted the identified phages. Only two cultivable bacterial endosymbionts, BEV (E*ndobacterales* [40]) and Asaia sp. (*Rhodospirillales* [33,60]), are known to be facultatively associated with *E. variegatus*. Because comprehensive studies of the bacterial fraction of its microbiome had never been undertaken, we did not have any a priori knowledge about either the possibility of isolation in axenic culture of the other endosymbionts or the most suitable media for their cultivation. The chosen growth media, usually adopted to identify enteric bacteria or to isolate fastidious bacteria, were effective in isolating the EVP-1 host. Interestingly, the EVP-1 host was shown to be the already known *E. variegatus* endosymbiont BEV, i.e., *Candidatus* Symbiopectobacterium. Moreover, colony-PCR proved that BEV was also the host of the other two identified major capsid protein-coding genes. Interestingly, the best blastx hits of the EVP-3 major capsid protein was the one identified in *Candidatus* Symbiopectobacterium sp. Dall1.0 during a metagenomic study of *Diachasma alloeum*, a parasitoid of the apple maggot *Rhagoletis pomonella* [61]. Such result further supported the proposal made by Martinson et al. [35] that the genus “*Candidatus* Symbiopectobacterium” represents a monophyletic group of invertebrate host-associated microbes. With the huge production of whole genome sequencing data, it is known that most bacterial genomes carry multiple prophages, a phenomenon called polylysogeny [62]; however, inferred prophage sequences do not always correspond to active temperate phages [63]. The association of multiple active phages to BEV was confirmed by electron microscope observations and PCR reactions on the bacterial colonies. The intense phage lytic activity observed in vitro may explain the low viability of the bacterium in culture. It is likely that, after a few days in pure culture, most bacterial cells undergo phage lysis as a result of the massive production of podovirus-like particles. By contrast, neither viral particles nor transcripts that could be associated with podoviruses were identified in the insect microbiome. This may indicate that these are in the prophage status in the insect environment, while they switch to the lytic phase in plate culture condition. A transcript coding for a myovirus-like major capsid protein was retrieved during the RNA-seq analysis, even if myovirus-like particles were not observed in the viral partial purification obtained from the whole insect. Hence, we can suppose either that the phage is present at very low concentration in the insect body (below the detection limit) or that the particles were, for some reason, destroyed during the purification process. Nevertheless, on the one hand, some regulation mechanisms should operate in the insect to prevent the podovirus-like phage from producing the same massive release of viral particles observed in pure culture, which could potentially cause the lysis of the whole bacterial population. On the other hand, the growth of the BEV population within the insect could be kept under control by the action of EVP-1 and, maybe, EVP-3.

It is known that temperate phages have an important role in shaping microbial diversity and community structure; in fact, not only do they alter the biology of their hosts (i.e., regulating gene expression, introducing novel functions), but they also influence the surrounding hosts and non-host bacterial cells (i.e., entering the lytic cycle and killing susceptible bacteria, liberating intracellular contents used as nutrients by neighboring cells) [64,65]. Moreover, bacteriophage–bacteria interactions are considered by Refardt [66] as an ideal system for studying the competitive interactions within hosts. In particular, he considered the competition among phages in the same host, which is still an unexplored area in phage ecological research, and showed that multiple infection in *Escherichia coli* often resulted in a decreased lytic productivity.

At present, we do not know whether BEV active phages can influence the lysogenic status of the other prophages or whether they can infect other insect bacterial endosymbionts. These two phenomena deserve to be elucidated in view of using this system as a model both for among-hosts competition studies and for developing microbiome-based new plant pest control strategies. In fact, unravelling the microbiome of insect vectors and understanding the complex relationships within its components may help to reveal possible microbe influences on pathogen transmission, and it is a crucial step toward an innovative sustainable strategies for disease management in agriculture.

## 4. Materials and Methods

### 4.1. Insect Population

*Euscelidius variegatus* of the Torino (Italy) phytoplasma-free laboratory colonies (EvaTO) were originally collected in Piedmont (Italy) and reared on oat, *Avena sativa* (L.), inside plastic and nylon cages in growth chambers at 20–25 °C with a L16:D8 photoperiod.

### 4.2. DNA and RNA Extraction

DNA and total RNA were extracted from either whole bodies or dissected organs of emerged EvaTO adults, as described by Marzachì et al. [67] and Ottati et al. [68], respectively.

In order to distinguish the encapsidated phage DNA (lytic infection) from the phage DNA integrated into the host chromosome (lysogenic infection), we proceeded as follows. Bacterial colonies were resuspended in sterile water and filter-sterilized with a 0.22 µm filter. Such step should eliminate bacterial hosts and enrich the aqueous phase with phage particles. The suspension was then treated with TURBO™ DNase (Thermo Fisher Scientific, Waltham, MA, USA) for 1 h at 37 °C to digest any residual free DNA and subjected to DNA extraction with one volume of phenol:chloroform:isoamyl alcohol (25:24:1 *v*/*v*) followed by a wash step with one volume of chloroform:isoamyl alcohol (24:1 *v*/*v*) to remove any trace of phenol. Finally, DNA was precipitated with sodium acetate/ethanol, washed with 70% ethanol, and resuspended in 10 mM Tris-HCl pH 8.2. The same DNase treatment and DNA extraction protocol were also used on semipurified viral particles.

### 4.3. RNA-Seq and Bioinformatic Analysis

Six micrograms of total RNA were sent to Macrogen (Seoul, Korea) for cDNA library construction and sequencing, as detailed in [36]. At least 100 million 100-nt paired-end reads were obtained for each dataset. The two datasets were merged and the pre-assembly steps were performed using BBTools suite v38.70, as previously described [11]. BBMap, in particular, was used to remove reads mapping to the *Euscelidius variegatus* transcriptome shotgun assembly (TSA Accession number: GFTU00000000.1) before the assembling step with Trinity v2.9.1 [69]. The resulting sequences were further assembled by CAP3 v3 (overlap length cutoff = 60; overlap percent identity cutoff N = 90) [70]. As a result, around 120,000 assembled sequences were obtained and queried against the NCBI non redundant “nr” protein database (Last access February 2021) with DIAMOND v0.9.24.125 [71] using an E-value cut-off of 0.0001. Bowtie2 [72] was used to map reads against the putative phage transcripts with default parameters. Reads mapping onto the selected transcripts were expressed as RPK (Reads Per Kilobase of transcript).

ViPTree [37] was used to automatically generate the annotation of the selected transcripts. Given that the tool used the RefSeq release 93, the predicted coding sequences and the corresponding deduced proteins were analyzed using NCBI RefSeq Release 205 (February 2021).

### 4.4. Accession Numbers

Reads were deposited into the NCBI’s Sequence Read Archive (SRA) database with BioSample accessions SAMN18744878 and SAMN18744879 as part of BioProject PRJNA393620. The partial 16S ribosomal RNA sequence of *Candidatus* Symbiopectobacterium strain EvaTO and the 12 phage transcripts were submitted to NCBI GenBank with accessions MW936016 (16S rRNA) and MW965281 MW965292 (phage sequences).

### 4.5. Phylogenetic Analysis

Phylogenetic relationships were inferred on the basis of the amino acid sequences of phage major capsid proteins. The three newly identified major capsid proteins were aligned with MUSCLE [73] to their best blastx hits and the first ten hits identified by blastp analysis against the NCBI RefSeq protein limited to the taxon “Viruses (taxid:10239)”. Phylogenetic trees were then generated using the maximum likelihood (ML) approach, implemented in IQ-TREE [74] with default parameters through the CIPRES Science Gateway V. 3.3 [75]. Bootstrap analyses involving 1000 replicates were used with the Dayhoff substitution matrix to estimate the pairwise distances.

### 4.6. PCR and RT-PCR Amplifications

PCR was used to verify the presence of the three genes, encoding the major capsid proteins in EvaTO insects, phage DNA, and bacterial colonies. Universal 16S rRNA bacterial primers were first used, and then BEV3/BEV4 primers were used to identify the bacterial host harboring EVP-1 phage by colony PCR. BEV primers were also used to exclude the presence of residual bacterial host DNA whenever it was necessary to distinguish the encapsidated phage DNA from the phage DNA integrated into the bacterial chromosome.

For each sample, cDNA was synthesized from total RNA (500 ng) with EvaTO_phage1r primer using the RevertAid Reverse Transcriptase (Thermo Fisher Scientific, Waltham, MA, USA). The absence of contaminating genomic DNA was verified including, in the PCR step, samples without the reverse transcription step.

All primer sequences and amplification conditions used in this work are listed in Table 2. The resulting amplicons were validated by Sanger sequencing at BMR Genomics (Padua, Italy).

**Table 2:** List of Primers Used in this Work.

Primer Name	Sequence	Target	Product Size (bp)	Annealing T (°C)	Citation
EvaTO_phage1f	CCGGTGGGTTCACTTTCC	MW965291	697	64	This work
EvaTO_phage1r	CGTCCGCAGACCATTATCGG				
EvaTO_phage2f	CTTCTCTGGCTGGCCTACCC	MW965292	725	64	This work
EvaTO_phage2r	GAGTATCGCCGGTCATCACG				
EvaTO_phage3f	AGGGTACTAGCCAGGACGAC	MW965289	524	64	This work
EvaTO_phage3r	TGTGCCGCCATTTCGATAAG				
BEV3	TTATGAGGTCCGCTTGCTCT	BEV 16S ribosomal DNA sequence	1009	64	[39]
BEV4	CGATCCCTAGCTGGTCTGAG				
27F	AGAGTTTGATCMTGGCTCAG	16S ribosomal DNA sequence	1507	58	[76]
1494R	CTACGGCTACCTTGTTACGA				

### 4.7. Bacterial Isolation

CO_2_-anesthetized leafhoppers were surface-sterilized by submerging them first in 95% ethanol for 1 to 2 min, then in 1.2 to 1.5% sodium hypochlorite solutions for 2 min and, eventually, rinsing them 2 or 3 times in sterile water [40]. Under a dissecting microscope, the hemolymph was aspirated with a fine, flame-drawn needle inserted into the insect body, between the thorax and the abdomen. The fluid was transferred to a tube containing 1X PBS. The hemolymph of five individuals was combined, then split in two and plated onto chocolate (Blood Agar Base—Sigma Aldrich—added with 7% horse defibrinated blood) and purple agar (Bromocresol Purple Broth—Sigma Aldrich—added with 1.5% agar) solid medium. The hemolymph of a total of 40 individuals was obtained. Plates were kept at 26 °C in the dark for up to 14 days. Colonies were numbered (using C when isolated from chocolate agar and P when isolated from purple agar plates) and transferred onto new chocolate and purple plates. For subcultures and maintenance, a Tryptic Soy Agar (TSA) medium (Sigma Aldrich) was eventually used.

### 4.8. Transmission Electron Microscopy

Viral particles were partially purified following the protocol previously described by [36]. Insect guts were collected under a dissecting microscope and crushed in 0.1 M phosphate buffer pH 7, added with 2% PVP, to obtain a crude extract. A portion of the bacterial colonies was collected with a toothpick from cultures on solid medium plates and suspended in 20 μL of liquid growth medium. A drop of the viral partial purification, the gut crude extract, or the bacterial suspension was deposited on carbon and formvar coated copper-palladium grids and left to stand for about 3 min. Grids were washed with water and negatively stained with 0.5% aqueous uranyl acetate. Observations and image acquisition were done using a CM 10 electron microscope (Philips, Eindhoven, The Netherlands) operating at 80kV. Micrograph films were developed and then digitally acquired at high resolution with a D800 Nikon camera. Images were trimmed and adjusted for brightness and contrast using GIMP 2 software. Particle measurements were done using Fiji software.

## Figures and Tables

**Table 1:** ViPTree Gene ORF Prediction and Blastx Analysis of the 12 Selected Phage Sequences.

Transcripts IDs and Predicted ORFs	Length (bp)	Range	RPK	Hit Description [Organism]	% Identities	E-Value	% Query Coverage
MW965290	5544		111.7				
ORF1	398	1–398		WP_195316289.1 phage tail tape measure protein [*Serratia marcescens*]	70	2 × 10^−51^	97
ORF2 #	357	398–754		WP_039351538.1 phage tail protein [*Pectobacterium fontis*]	90	1 × 10^−75^	97
ORF3 #	753	804–1556		MBG6243408.1 phage minor tail protein L [*Candidatus* Symbiopectobacterium sp. Dall1.0]	96	0.0	100
ORF4 #	576	1713–2288		MBG6243407.1 peptidase P60 [*Candidatus* Symbiopectobacterium sp. Dall1.0]	95	8 × 10^−165^	94
ORF5 #	606	2272–2877		WP_104212022.1 tail assembly protein [*Pectobacterium brasiliense*]	81	2 × 10^−85^	100
ORF6	2611	2934–5544		WP_104212026.1 phage tail protein [*Pectobacterium brasiliense*]	91	2 × 10^−70^	100
MW965281	2188		112				
ORF1	318	255–572		WP_021179416 fimbrial protein TcfA [*Serratia fonticola*]	62	4 × 10^−29^	90
ORF2 #	252	557–808		WP_146751463.1 ANR family transcriptional regulator [*Enterobacter cloacae* complex]	56	5 × 10^−7^	60
ORF3 #	1089	859–1947		WP_187497555.1 phage tail protein [*Pantoea* Psp39-30]	43	2 × 10^−82^	98
MW965289	1777		128.3				
ORF1	99	1–99		EFC4054519.1 HK97 family phage prohead protease [*Escherichia coli*]	81	2 × 10^−9^	100
ORF2 #	1227	109–1335		MBG6243159.1 phage major capsid protein [*Candidatus* Symbiopectobacterium sp. Dall1.0]	85	0.0	99
ORF3 #	300	1426–1725		WP_044208854.1Phage gp6-like head-tail connector family protein [*Pectobacterium odoriferum*]	87	3 × 10^−57^	100
MW965291	6115		213.4				
ORF1	580	1–580		WP_108703399 Terminase small subunit [*Enterobacter hormaechei*]	96	2 × 10^−86^	96
ORF2 #	1659	583–2241		WP_108703400 terminase large subunit [*Citrobacter europaeus*]	98	0.0	100
ORF3 #	1935	2324–4258		WP_135684645.1 phage major capsid protein [*Klebsiella pneumoniae*]	95	0.0	99
ORF4 #	168	4297–4464		WP_181941880.1 hypothetical protein [*Klebsiella pneumoniae*]	89	8 × 10^−28^	100
ORF5 #	1359	4464–5822		NIC64170.1 phage portal protein [*Klebsiella pneumoniae*]	94	0.0	100
ORF6	297	5819–6115		RTO54147.1 phage gp6-like head-tail connector protein, partial [*Enterobacter hormaechei*]	80	8 × 10^−47^	100
MW965287	1419		71.2				
ORF1	482	1–482		MBD2797976.1 HK97 family phage prohead protease [*Xenorhabdus* sp. 18]	72	6 × 10^−74^	96
ORF2	953	467–1419		QBY47020.1 phage portal protein [*Arsenophonus nasoniae*]	92	0.0	100
MW965282	770		61.0	WP_187497555.1 putative phage tail protein [*Plautia stali* symbiont]	99	1 × 10^−158^	84
MW965283	3268		114.1	WP_113869621.1 phage tail tape measure protein [*Brenneria salicis*]	82	0.0	82
MW965286 *	251		47.8	SPW64604.1 putative head-tail adaptor [*Escherichia coli*]	98	1 × 10^−20^	73
MW965288	768		50.8	MBJ9599707.1 phage portal protein [*Citrobacter werkmanii*]	99	4 × 10^−179^	94
MW965292 #	1852		123.1	WP_164114194.1 phage major capsid protein [S*erratia marcescens*]	63	0.0	77
MW965285	299		23.4	WP_010281992.1 portal protein [*Pectobacterium brasiliense]*	95	8 × 10^−55^	92
MW965284 *	345		46.4	SUH06759.1 portal protein [*Salmonella enterica* subsp. enterica]	87	1 × 10^−27^	66

RPK: read counts divided by the length of each transcript in kilobases. * putative pseudogene (presence of frameshifts). # Complete CDS. Grey shaded cells represent different transcript IDs.

## Data Availability

Reads were deposited into the NCBI’s Sequence Read Archive (SRA) database with BioSample accessions SAMN18744878 and SAMN18744879 as part of BioProject PRJNA393620. The partial 16S ribosomal RNA sequence of *Candidatus* Symbiopectobacterium strain EvaTO and the 12 phage transcripts were submitted to NCBI GenBank with accession MW936016 (16S rRNA) and MW965281—MW965292 (phage sequences).

## Supplementary Materials

The following are available online at https://www.mdpi.com/article/10.3390/pathogens10050612/s1, Figure S1: (**a**) Examples of chocolate agar plates with colonies grown after plating hemolymph extracted from *Euscelidius variegatus* Torino population. (**b**) List and characteristics of all the isolated colonies from chocolate (C) and purple (P) agar plates; positivity to EVP-1 was determined by PCR with EVP-1 primers. Figure S2: Alignment of the 16S ribosomal sequence of the three colonies with the one from the “Bacterial parasite of *Euscelidius variegatus*” (BEV) GenBank accession number: Z14096; (Campbell and Purcell, 1993). Table S1: Blastn analysis of the 12 selected phage sequences against the BEV sequences submitted to the GenBank Trace Archive.
