# Supplementary material for: Bacteriophage-Host Association in the Phytoplasma Insect Vector Euscelidius variegatus
Source: Pathogens. 2021 May 17;10(5):612. doi: 10.3390/pathogens10050612 (PMC8156552; doi:10.3390/pathogens10050612)
Supplement: Supplementary file 1 [file pathogens-10-00612-s001.zip › pathogens-1220601-supplementary.pdf]

Supplementary Materials

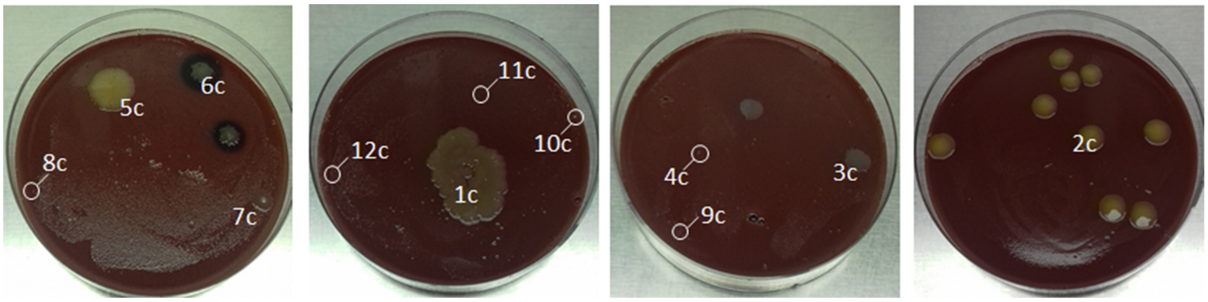

(a)

Colony name	Day of appearence	Colony aspect	Positive for EVP-1
1C	1-3	pale yellow, irregular, flat, undulate margin	
2C	1-3	bright yellow, circular, raised, smooth margin	
3C	1-3	white, irregular, flat, undulate margin	
4C	7-10	white transparent, circular, tiny	x
5C	1-3	bright yellow, circular, raised, irregular margin	
6C	1-3	white, irregular, flat, lobate margin, black halo	
7C	1-3	white, irregular, umbonate, lobate margin,	
8C	7-10	white transparent, circular, tiny	x
9C	7-10	white transparent, circular, tiny	x
10C	7-10	yellow, circular, tiny	
11C	7-10	white transparent, circular, tiny	
12C	7-10	white transparent, circular, tiny	x
13C	7-10	white transparent, circular, tiny	x
14C	7-10	white transparent, circular, tiny	x
15C	7-10	white transparent, circular, tiny	x
1P	1-3	orange, circular, raised, lobate margin	
2P	1-3	pale yellow, irregular, flat, smooth margin	
3P	7-10	white transparent, circular, tiny	x
4P	7-10	white transparent, circular, tiny	x
5P	7-10	white transparent, circular, tiny	x

(b)

Figure S1. (a) Examples of chocolate agar plates with colonies grown after plating hemolymph extracted from *Euscelidius variegatus* Torino population. **(b)** List and characteristics of all the isolated colonies from chocolate (C) and purple (P) agar plates; positivity to EVP-1 was determined by PCR with EVP-1 primers.

```
Z14096.1        ----------CATGGCTCAGATTGAACGCTGGCGGCAGGCCTAACACATGCAAGTCGAGC     50
16S_colony      AGAGTTTGATCATGGCTCAGATTGAACGCTGGCGGCAGGCCTAACACATGCAAGTCGAGC     60
                          **************************************************

Z14096.1        GGTAGCACAAGGGAGCTTGCTCCCCGGGTGACGAGCGGCGGACGGGTGAGTAGTGTCTGG     110
16S_colony      GGTAGCACAAGGGAGCTTGCTCCCCGGGTGACGAGCGGCGGACGGGTGAGTAGTGTCTGG     120
                ************************************************************

Z14096.1        GAAACTGCCTGATGGAGGGGGATAACTACTGGAAACGGTAGCTAATACCGCATAACGTCG     170
16S_colony      GAAACTGCCTGATGGAGGGGGATAACTACTGGAAACGGTAGCTAATACCGCATAACGTCG     180
                ************************************************************

Z14096.1        CAAGACCAAAGTGGGGGACCTTCGGGCCTCACGCCATCAGATGTGCCCAGATGGGATTAG     230
16S_colony      CAAGACCAAAGTGGGGGACCTTCGGGCCTCACGCCATCAGATGTGCCCAGATGGGATTAG     240
                ************************************************************

Z14096.1        CTAGTAGGTGGGGTAACGGCTCACCTAGGCGACGATCCCTAGCTGGTCTGAGAGGATGAC     290
16S_colony      CTAGTAGGTGGGGTAACGGCTCACCTAGGCGACGATCCCTAGCTGGTCTGAGAGGATGAC     300
                ************************************************************

Z14096.1        CAGCCACACTGGAACTGAGACACGGTCCAGACTCCTACGGGAGGCAGCAGTGGGGAATAT     350
16S_colony      CAGCCACACTGGAACTGAGACACGGTCCAGACTCCTACGGGAGGCAGCAGTGGGGAATAT     360
                ************************************************************

Z14096.1        TGCACAATGGGCGCAAGCCTGATGCAGCCATGCCGCGTGTGTGAAGAAGGCCTTCGGGTT     410
16S_colony      TGCACAATGGGCGCAAGCCTGATGCAGCCATGCCGCGTGTGTGAAGAAGGCCTTCGGGTT     420
                ************************************************************

Z14096.1        GTAAAGCACTTTCAGCGAGGAGGAAGGCAGTAAGGTTAATAACCTTGCTGATTGACGTTA     470
16S_colony      GTAAAGCACTTTCAGCGAGGAGGAAGGCAGTAAGGTTAATAACCTTGCTGATTGACGTTA     480
                ************************************************************

Z14096.1        CTCGCAGAAGAAGCACCGGCTAACTCCGTGCCAGCAGCCGCGGTAATACGGAGGGTGCAA     530
16S_colony      CTCGCAGAAGAAGCACCGGCTAACTCCGTGCCAGCAGCCGCGGTAATACGGAGGGTGCAA     540
                ************************************************************

Z14096.1        GCGTTAATCGGAATGACTGGGCGTAAAGCGCACGCAGGCGGTTTGTTAAGTTGGATGTGA     590
16S_colony      GCGTTAATCGGAATGACTGGGCGTAAAGCGCACGCAGGCGGTTTGTTAAGTTGGATGTGA     600
                ************************************************************

Z14096.1        AATCCCCGGGCTTAACCTGGGAAACTGCATTCAAAACTGGCAAGCTAGAGTCTCGTAGAG     650
```

```
16S_colony      AATCCCCGGGCTTAACCTGGG-AACTGCATTCAAAACTGGCAAGCTAGAGTCTCGTAGAG     659
                ********************* **************************************

Z14096.1        GGGGGTAGAATTCCAGGTGTAGCGGTGAAATGCGTAGAGATCTGGAGGAATACCGGTGGC     710
16S_colony      GGGGGTAGAATTCCAGGTGTAGCGGTGAAATGCGTAGAGATCTGGAGGAATACCGGTGGC     719
                ************************************************************

Z14096.1        GAAGGCGGCCCCCTGGACGAAGACTGACGCTCAGGTGCGAAAGCGTGGGGAGCAAACAGG     770
16S_colony      GAAGGCGGCCCCCTGGACGAAGACTGACGCTCAGGTGCGAAAGCGTGGGGAGCAAACAGG     779
                ************************************************************

Z14096.1        ATTAGATACCCTGGTAGTCCACTCTGTAAACGATGTCGATTTGGAGGTTGTGCCCTTGAG     830
16S_colony      ATTAGATACCCTGGTAGTCCACGCTGTAAACGATGTCGATTTGGAGGTTGTGCCCTTGAG     839
                ********************** *************************************

Z14096.1        GCGTGGCTTCCGGACGTAACGCGTTAAATCGACCGCCTGGGGGAGTACGGCCGCAAGGTT     890
16S_colony      GCGTGGCTTCCGGAGCTAACGCGTTAAATCGACCGCCTGGGG-AGTACGGCCGCAAGGTT     898
                **************  ************************** *****************

Z14096.1        AAAAGTGAAATGAATTGACGGGG-CCGCTACAAGCGGTGGAGCATGTGGTTTAATTCGAT     949
16S_colony      AAAACTCAAATGAATTGACGGGGGCCCGCACAAGCGGTGGAGCATGTGGTTTAATTCGAT     958
                **** * **************** **   *******************************

Z14096.1        GCAACGCGAAG-ACCTTACCTACTCTTGACATCCAGAGAATTTAGCAGAGATGCTTTAGT     1008
16S_colony      GCAACGCGAAGAACCTTACCTACTCTTGACATCCAGAGAATTTAGCAGAGATGCTTTAGT     1018
                *********** ************************************************

Z14096.1        GC-TTCGGGAACTCTGAGACAGGTGCTGCATGGCTGTCGTCAGCTCGTGTTGTGAAATGT     1067
16S_colony      GCCTTCGGGAACTCTGAGACAGGTGCTGCATGGCTGTCGTCAGCTCGTGTTGTGAAATGT     1078
                ** *********************************************************

Z14096.1        TGGGTTAAGTCCCGCAACGAGCGCAACCCTTATGCTTTGTTGCCAGCGATTCGGTCGGGA     1127
16S_colony      TGGGTTAAGTCCCGCAACGAGCGCAACCCTTATGCTTTGTTGCCAGCGATTCGGTCGGGA     1138
                ************************************************************

Z14096.1        ACTCAAAGGAGACTGCCAGTGATAAACTGGAGGAAGGTGGGGATGACGTCAAGTCATCAT     1187
16S_colony      ACTCAAAGGAGACTGCCAGTGATAAACTGGAGGAAGGTGGGGATGACGTCAAGTCATCAT     1198
                ************************************************************

Z14096.1        GGCCCTTACGAGTAGGGCTACACACGTGCTACAATGGCGTATACAAAGAGAAGCGACCGC     1247
16S_colony      GGCCCTTACGAGTAGGGCTACACACGTGCTACAATGGCGTATACAAAGAGAAGCGACCTC     1258
```

```
                ********************************************************** *

Z14096.1        GCGAGAGCAAGCGGACCTCATAAAGTACGTCGTAGTCCG-ATTGGAGTGTGCAACTCGAC     1306
16S_colony      GCGAGAGCAAGCGGACCTCATAAAGTACGTCGTAGTCCGGATTGGAGTCTGCAACTCGAC     1318
                *************************************** ******** ***********

Z14096.1        TCCATGAAGTCGGAATCGCTAGTAATCGTAGATCAGAATGCTACGGTGAATACGTTCCCG     1366
16S_colony      TCCATGAAGTCGGAATCGCTAGTAATCGTAGATCAGAATGCTACGGTGAATACGTTCCCG     1378
                ************************************************************

Z14096.1        GGCCTTGTACACACCGCCCGTCACACCATGGGAGTGGGTTGCAAAAGAAGTAGGTAGCTT     1426
16S_colony      GGCCTTGTACACACCGCCCGTCACACCATGGGAGTGGGTTGCAAAAGAAGTAGGTAGCTT     1438
                ************************************************************

Z14096.1        AACCTTCGGGAGGGCGCTTACCACTTTGTGATTCATGACTGGGGTGAAGTCGTAACAAGG     1486
16S_colony      AACCTTCGGGAGGGCGCTTACCACTTTGTGATTCATGACTGGGGTGAAGTCGTAACAAGG     1498
                ************************************************************

Z14096.1        TAACCGTAGGGG------------------------------------------------     1498
16S_colony      TAACCGTAGGGGAACCTGCGGTTGGATCACCTCCTTACCTAATGATACTGATTCTGTGAA     1558
                ************

Z14096.1        ----- 1498
16S_colony      GTGTT 1563
```

Figure S2. Alignment of the 16S ribosomal sequence of the three colonies with the one from the "Bacterial parasite of *Euscelidius variegatus*" (BEV) GenBank accession number: Z14096; (Campbell and Purcell, 1993)

Table S1. Blastn analysis of the 12 selected phage sequences against the BEV sequences submitted to the GenBank Trace Archive

Trasncript IDs	Length (nt)	hit accession number	hit length	E-value	identities %	query coverage %
MW965288	768	gnl|ti|2292004869	1322	0	98	94
MW965287	1419	gnl|ti|2292004924	1177	0	100	63
MW965291	6115	gnl|ti|2292005037	1289	0	99	17
MW965289	1777	gnl|ti|2292005093	1272	0	100	38
MW965290	5544	gnl|ti|2292005198	1211	6E-176	100	6
MW965282	770	gnl|ti|2292005213	1160	2E-157	97	42
MW965292	1852	gnl|ti|2292005268	1315	0	88	42
MW965284	345	gnl|ti|2292005461	1125	2E-31	95	23
MW965286	251	gnl|ti|2292005461	1125	5E-50	98	43
MW965283	3268	gnl|ti|2292005498	1266	0	99	32
MW965281	2188	gnl|ti|2292005561	1072	0	98	46
MW965285	299	No hits found				
